# Local Anæsthesia

**Published:** 1856-04

**Authors:** H. L. Burpee

**Affiliations:** 35 Fourth-st., Brooklyn, E. D.


### ARTICLE XX.

### *Local Anæsthesia.*

Editor Dental Recorder:

*Dear Sir:*—Since the issue of the December number of your valuable journal, containing some account of J. Richard Quinton's experience with anæsthesia by congelation, I have labored almost day and night to perfect an apparatus, by which a cold fluid could be successfully applied for the extraction of teeth.

I little thought, when I drew a plan upon paper, that it would require several weeks to get into operation, but, let the requisite amount of time be what it may, it was not ready for use in my hands, with all I could learn from the truly valuable and interesting account referred to, until about the 15th of the present month (Feb.,) although I had submitted to what I considered a successful application in my own mouth some weeks sooner.

I have found much difficulty in the preparation of an apparatus that will prevent the flow of fluid in the mouth (and still be applicable to tooth and gum.) This having at length been successfully accomplished, I found myself under some embarrassment to find some person who was willing to allow me to experiment upon teeth, by extracting them when I thought I had produced the requisite loss of sensation. In this I at length succeeded, by offering a reward to a lady who was once above want but now obliged to sew for a livelihood, and who was dreading, as well, the expense of artificial teeth, as the removal of a quantity of old stumps and decayed teeth. By this means, I have had ample opportunity to experiment in the patient's mouth, not only upon the anæsthesia, but the improvement of the apparatus.

In the early part of my experiments, I found that something different from an elastic tube, with the flow regulated by stop cocks, was required for general use. Although such an apparatus could, in some cases, be used with an assistant (which, although provided with one myself at present, I have very good reason to suppose dentists are not very generally so fortunate.) Hence my object has been so to adapt the instrument that it could come into general use, and even be applied by one acquainted, in his own mouth. This I have so nearly accomplished, as to have one ready and now in use by myself, and as soon as I can get the apparatus made in any quantity, I shall be willing to demonstrate the *modus operandi* to the profession, should it be desirable. I am convinced, from my own experience, that the principle as laid down by Quinton, is, in fact, applicable in dental surgery; but I am not fully convinced as to the specific degree of cold necessary to use. On this point I am still experimenting; the result of my experience, thus far, I would give at this time, were I not prevented from want of time. So far as my experience goes with the use of this new agent, I am highly gratified with the result, as it has placed the matter on this ground,—*that it can be successfully employed by competent hands.* The fluid thus far used by myself was known in my "school-boy days," but I think I have, in some instances, used a colder fluid than necessary; but in this matter much depends upon the mode of application. But as there is no danger in experiments, that matter can be easily determined so far as to insure success.

H. L. Burpee.

35 *Fourth-st., Brooklyn, E. D.* [*Dent. Rec.*

